# Intelligence

**Published:** 1810-05

**Authors:** 


### INTELLIGENCE.

The late Army Medical Board, as was generally expected, has been dissolved, and a new one appointed, consisting of Mr. Weir, Drs. Kerr, and Theodore Gordon. This arrangement seems to be no ways pleasing to Sir Lucas Pepys, from whom it has called forth the following letter, which we present to our readers, as a specimen of the *Zeal and Ardour* with which Sir Lucas contends for the *exclusive* appointment of the medical graduates of Oxford and Cambridge to the Army, whom, with no great degree of liberality, he designates as the *only regular physicians*, although he acknowledges those Universities are not medical schools. The Secretary at War would not say whether he thought a *previous* academical education is necessary or adviseable, before the commencement of medical studies. It cannot be necessary to the welfare of the army, if medical knowledge cannot be attained by it.

On the late establishment, all the physicians to the army were obliged to be examined by the College, of which Sir Lucas is president; no one, therefore could be approved, who was not *qualified* to undertake the cure of diseases; and of what consequence could it be to the sick soldier, whether the physician who cured him, was or was not a Fellow of the College? In point of general literature, which is confessedly so ornamental to a physician, we think there are few graduates of Edinburgh who could not, at least, *equal* Sir Lucas's Letter in elegance of diction and perspicuity of style, although he had never resided in an English University, an advantage Sir Lucas so exultingly boasts of.

*Copy of a Letter from the Physician General to the Secretary at War.*

"My Lord,

"Cambridge, Oxford, and Dublin Universities will have to lament your Lordship's acquiescence in the lessening of the credit and character of one of the learned professions. The regular physicians from those Universities were gradually getting into the charge of the army! This I have been bringing about for the last 16 years; and when I first came into the army, there was only one regularly educated physician in it. Now there are five Fellows of the College of Physicians, and only two out of the whole number who have not been approved by the College. The Board about to be established under your Lordship's sanction, propose to have the care of the army committed entirely to regimental surgeons. Whether your Lordship was of opinion, that previous academical education is necessary, or adviseable, before the study of medicine in the medical schools, either here or at Edinburgh, I wished to know when I had the honour of waiting upon your Lordship lately; but as I found you declined to enter on the subject, I thought it my duty, as President of the College of Physicians, and well known by the Universities as a zealous advocate for academical education, to trouble your Lordship with this, that it might hereafter be known that I had expressed my opinion in writing on this important subject.

ject. A Board of General Officers determining on the medical arrangements of the army, is not* more incompetent than a College of Physicians to decide on the attack of a fortress. As the Commander in Chief disavows the intended change, as originating with him, your Lordship will see the propriety of my addressing you, before it is too late, to request that the abolition of regularly educated physicians from the army may be well considered by your Lordship. For this purpose, I hope my paper delivered to Mr. Percival, on the subject of the fifth Report, two years ago, has been read by your Lordship, and also the Appendix, No. 8, B 4 and 5, of Mr. Keate's's printed observations, where your Lordship will see the consequence when surgeons, called physicians, but really not so, act as physicians. I can have no other inducement in troubling your Lordship with this, but my attachment to the Universities, and a sincere wish for the welfare of the army.

"L. PEPYS.

"Upper Brook Street, Jan. 28, 1810."

---

Mr. Luke Howard, of Plaistow, has detected a criminal imposition, the knowledge of which cannot be too widely circulated, or its effects too carefully guarded against. A very large quantity of glass of lead, has, by some means, found its way into the London market, as glass of antimony. This imposition is sure to be discovered in the operation to which the latter is chiefly applied, the making of emetic tartar; but it is highly necessary for the consumers of smaller quantities, as in the *vitrum ceratum* and *vitrum antimonii*, to be acquainted with the following distinctive characters of the two, that those who have bought the article within the last twelve or eighteen months, may assure themselves of its being genuine. The public health, and even the lives of many patients, may be considered at stake on this occasion. Glass of antimony has a rich brown, or reddish colour, with the usual transparency of coloured glasses. The glass of lead is of a deeper and duller colour against the light, is much less transparent, and even in some samples quite opaque. The specific gravity of the true, never exceeds 4·95, that of the spurious is 6·95: or in round numbers their comparative weights are as 5 to 7. Let twenty grains be rubbed fine in a glass mortar, adding half an ounce of good muriatic acid. The true dissolves with an hepatic smell; the solution is turbid, but has no sediment. The spurious turns the acid yellow, giving out an oxymuriatic odor, and leaves much sediment. Let a little of each solution be separately dropped into water. The true deposits oxyde of antimony, in a copious white coagulum; or, if the water has been previously tinged with sulphuret of ammonia, in a fine orange precipitate. The spurious gives no precipitate in water, and in the other liquid, one of dark brown, or olive colour. A solution of the spurious in vinegar has a sweet taste, together with the other properties of acetate of lead. A very small mixture of

---

* We presume Sir Lucas means less incompetent.

of it may be detected, by its debasing, more or less, the bright orange colour of the precipitate thrown down by sulphuret of ammonia, from the solution in any acid. The samples of the spurious hitherto detected, are of a much thicker and clumsier cast than the genuine; but the appearance is not to be trusted, and no specimen should be allowed to pass without a trial, either of the specific gravity, or chemical properties.

On an examination after death of the man who was wounded on Tower Hill, by the soldiers, on the 9th ult. and carried into the London Hospital, it was discovered that the ball had entered the fore part of the neck, had detached one of the alæ of the thyroid cartilage, passed clear of the carotid artery, and wounded the vertebral; it had afterwards fractured the transverse process of one of the cervical vertebræ, and finally lodged under the integuments at the back part of the neck The poor man survived only three days.

Dr. ADAMS's Lectures on Pathology and the Practice of Medicine, will commence on the 27th inst. at ten o'clock in the morning. —During that part of the Course which embraces Morbid Poisons, all the varieties of Vaccination and Small-pox, casual and inoculated, will be exhibited at the Hospitals.

Dr. CLUTTERBUCK will begin his Summer Course of Lectures on the Theory and Practice of Physic, Materia Medica, and Chemistry, on Monday June the 4th, at a quarter before ten in the morning, at his house, No. 1, Crescent, New Bridge Street, where further particulars may be had.

Dr. JAMES ROBERTON, Member of the College of Physicians, and one of the Physicians to the British Lying-in Hospital, will begin his Summer Course of Lectures on the Principles and Practice of Midwifery, and the Diseases of Women and Children, about the 15th of May.—The Lectures will be delivered every morning, at Mr. Bell's Museum, No. 10, Leicester Street, Leicester Square.

We understand that Mr RAMSDEN is about to publish some important Cases of Cure of the derangements of the Testicle, demonstrative of their being sympathetic with the Urethra; and to shew that most of the Diseases of that Gland, hitherto deemed incurable, are perfectly within remedy. Also some Cases of Hydrocele, in which the radical Cure has been effected without recourse to any of the operations at present practised for that purpose.

